# Effect Evaluation of a Web-Based Coaching Intervention to Support Implementation of Sex Education Among Secondary School Teachers: Randomized Controlled Trial

**DOI:** 10.2196/jmir.7053

**Published:** 2018-06-18

**Authors:** Lisette Schutte, Fraukje E.F Mevissen, Suzanne Meijer, Theo Paulussen, Pepijn van Empelen, Gerjo Kok

**Affiliations:** ^1^ Department of Work and Social Psychology Maastricht University Maastricht Netherlands; ^2^ Department of Youth STI AIDS Netherlands Amsterdam Netherlands; ^3^ Expertise Group Child Health Netherlands Organisation for Applied Scientific Research Leiden Netherlands

**Keywords:** sex education, randomized controlled trial, coaching, secondary schools

## Abstract

**Background:**

The quality of implementation is important to ensure the effectiveness of behavioral change interventions in practice. Implementing such programs with completeness and adherence is not an automatic process and may require additional support. In school settings, the support teachers receive during implementation is often limited and appears to fall short when attempting to preserve completeness and adherence in program delivery. With the aim to improve completeness and adherence of teachers’ delivery of a sexual health promoting intervention (“Long Live Love” [LLL]) in secondary education, a Web-based e-coach was developed (“lesgevenindeliefde.nl”or“teachinglove.nl”). The effectiveness of the e-coach, as part of a broader implementation strategy, in influencing teachers’ implementation was evaluated.

**Objective:**

This study aimed to report on the effect evaluation to determine the effect of the Web-based e-coach on teacher implementation of a school-based sex education program called LLL and on its determinants.

**Methods:**

A cluster randomized controlled trial (e-coaching vs waiting list control) was conducted with a baseline assessment (T0) and follow-up (T1) 2 weeks after completing the LLL program. A total of 43 schools with 83 teachers participated in the study. In the follow-up, 38 schools participated, 23 in the e-coaching condition with 41 teachers and 15 in the control condition with 26 teachers. Multilevel regression analysis was used to evaluate the effect of the e-coaching website on implementation behavior, namely, completeness and adherence to LLL implementation, and on its determinants.

**Results:**

The e-coaching intervention was not found to have an effect on teachers’ implementation behavior; teachers assigned to the experimental e-coaching website did not score higher on completeness (*P*=.60) or adherence (*P*=.67) as compared with teachers in the control condition. When comparing the 30 teachers who made actual use of the e-coaching website with the 37 teachers who did not, no significant differences were found either (*P*≥.54). In addition, there was no effect of e-coaching on the determinants of teacher implementation behavior (t_67-75_≤0.69; *P*≥.22).

**Conclusions:**

E-coaching was not found to be effective in enhancing completeness of and adherence to LLL by teachers. The lack of effect may be attributed to the intervention content, the limited use, or the study design itself. The e-coaching intervention may not have adequately addressed adherence and completeness of LLL to bring about behavioral change. Furthermore, the e-coaching intervention was not or insufficiently used by teachers. A possible biased sample of motivated, able teachers may have agreed to participate in the study, and a possible “ceiling effect” may have been present because of the high implementation grade. This, however, does not imply that Web-based coaching in itself is an ineffective strategy to promote adherence and completeness of program implementation. A process evaluation is required as follow-up.

**Trial Registration:**

International Standard Randomised Controlled Trial Number ISRCTN11754581; http://www.isrctn.com/ISRCTN11754581 (Archived by WebCite at http://www.webcitation.org/70C5TUOOh)

## Introduction

### Background

Implementation is important to ensure the effectiveness of an intervention in practice. An intervention that is implemented completely and according to its guidelines is more likely to be successful in changing the target groups’ determinants and behavior than programs that are not implemented fully [1-3]. In school-based sexual health promotion, teachers are the key players for the implementation of these programs. Their implementation is, however, often suboptimal; programs are not being implemented completely or with sufficient fidelity to produce measurable outcomes [4-8]. Several terms are used interchangeably to describe the fidelity of implementing an intervention. Fidelity has different dimensions or aspects, namely, the degree to which an intervention is conducted: (1) competently (competence) and (2) according to protocol (adherence). Adherence refers to the extent to which a program is implemented conforming to the guidelines. Competence relates to skillfulness in the delivery of the program, and thus how well it is implemented [3]. There is a need for greater attention to the quality of implementation and its related determinants, namely, teachers’ beliefs about the innovation and characteristics of the innovation, organizational factors, and characteristics of the implementation-enhancing intervention [9,10,6].

### Supporting Teachers During Implementation

Teachers appear to be in need of support in every phase of the implementation process to enable them to put the innovation into practice [3,11,12]. Supporting teachers in the implementation phase has, however, been insufficiently considered, as most work has been invested to promote teachers’ awareness and adoption of new interventions [4,13,9,14]. This applies in particular to school-based sex education programs, which address the sensitive subject of sexuality. Providing support before implementation in the form of training often equips teachers with skills for correct implementation, but this is not enough [15]. It remains important to provide teachers with more personal assistance and ongoing support and consultation during the process of putting an innovation into practice [6,16,17,14]. Currently, this support is limited to providing practical support in the form of teacher manuals with practical information on the content of the lessons and on how to deliver such lessons. However, more in-depth coaching focusing on determinants of implementation such as self-efficacy and social support to enhance completeness and fidelity is lacking [18,19,11,20-26].

### Web-Based Coaching to Improve Teachers’ Implementation of School-Based Sex Education

To stimulate the correct implementation, with completeness and adherence, of the (revised) school-based sex education program “Long Live Love” (LLL) [27,6], an e-coaching intervention (“lesgeveinindeliefde.nl” or “teachinglove.nl”) was systematically developed [28]. The e-coach aimed to improve teachers’ implementation behavior through self-reflection and skills development. Through e-coaching, we aimed at making teachers aware of the importance of completeness and adherence in relation to program effectiveness and increase their awareness regarding their own (suboptimal) implementation behavior. In addition, the e-coach provided tools to help teachers improve their implementation by giving support on how to deal adequately with potentially difficult classroom situations they could encounter when providing sexual and reproductive health (SRH) lessons, such as creating a safe atmosphere in the classroom for students to openly discuss relationships and sexuality, handling personal questions addressed to teachers by students, and intervening on negative remarks or behavior toward homosexuality. The content of the e-coach was guided by theories on implementation behavior [10,29] and based on a needs assessment among the target group [28]. Intervention objectives were psychosocial determinants, such as awareness, teachers’ personal benefit, social support, (anticipated) student responses, and self-efficacy. The e-coach could be used by teachers before and during deliverance of the LLL program. For a more detailed description of the e-coach, refer to the study by Schutte et al [28].

The e-coach was part of a broader strategy for implementation, aimed at promoting each phase of the LLL implementation process. The municipal health services (MHS) were involved in the development and delivery of the implementation strategy. This strategy included instruction protocols used by the MHS to promote adoption and continuation of LLL by teachers in schools, a teacher training delivered by the MHS, and a teacher manual to enhance and facilitate implementation. The MHS training was aimed at introducing the revised LLL program to teachers and motivating them to use the program and use it as intended by enhancing teachers’ knowledge, attitudes, and skills. The training was provided before implementation of LLL and was followed by e-coaching. An effect and process evaluation for the pilot implementation of the coaching website was conducted. This occurred simultaneously with the pilot implementation of the revised school-based LLL intervention for students [30]. The aim of this study was to determine the effect of e-coaching on (determinants of) teachers’ implementation behavior. The process evaluation is described elsewhere (unpublished data [31]).

## Methods

### Design

A clustered randomized controlled trial (e-coaching vs waiting-list control) was conducted, with a baseline assessment (T0) and follow-up (T1) 2 weeks after completing the LLL program. Teachers were not informed about the existence of these 2 groups.

### Recruitment and Procedure

From all the secondary schools in the Netherlands, 19.0% (115/610) of the schools were randomly selected after stratification according to region and education level (preparatory applied education, higher general continued education, and preparatory scholarly education). Teachers within these schools were invited by email and telephone to use the revised LLL program and to participate in a survey study on their experience and implementation of SRH education and LLL. Only teachers who taught SRH were contacted. In the Netherlands, teachers are the primary decision makers in the use of SRH programs [32]. The schools with teachers who accepted the invitation (n=45) were randomly assigned to either the control (n=20) or the intervention (e-coach) group (n=25).

Teachers in the intervention and control group who consented to participation first received the baseline survey (T0) by post. The T0 survey focused on determinants of SRH and LLL implementation and took approximately 30 min to fill out. Teachers had 2 weeks to complete and return the survey. Nonresponders got a reminder by email and eventually by telephone 3 days after the deadline, and were given another 2 weeks to return the survey.

At the same time, teachers in both groups were offered a training from the MHS in their region before implementing the revised LLL program. The training was offered to but not taken for personal reasons by 2 schools (4 teachers in the control group and 1 teacher in the intervention group). Finally, 39 of the participating teachers in the survey (24 from the intervention group and 15 from the control group) from 19 schools received training from 14 different MHS, as indicated at T1. The remaining teachers from 19 schools (11 teachers from the control group and 17 teachers from the intervention group) did not receive training, either because they refused the training as they felt there was no need or because the MHS in their region was not offering the training. Separate trainings were delivered to teachers in the e-coach intervention group versus the control group, with teachers in the intervention group receiving additional information during the training about the e-coaching website and being stimulated to use it during the implementation of LLL.

Teachers in both groups then received the LLL program (a package including a student magazine, a student DVD, and a teacher manual) by post mail, which they could implement within (approximately) 2 months following the baseline measurement for teachers (T0). Additionally, teachers in the intervention group were given access to the e-coaching website with a personal user name and password, and an edition of the LLL teacher manual, which contained references to the website. The teachers in the control group were not exposed to or informed about the website, until after the end of the e-coach evaluation. They received the regular LLL teacher manual without any references to the e-coach. Halfway during the pilot implementation, an email reminded teachers in the intervention group to use the e-coaching website. In addition, 1 week before the expected completion of the LLL program, all teachers were reminded by email and telephone about the upcoming posttest questionnaire (T1). Within 2 weeks after completing the implementation of the LLL program, the T1 survey was sent to all teachers. Reminders were sent by email and eventually by telephone to nonresponders. All procedures in the study were approved by the authorized Ethical Review Committee of Psychology & Neuroscience at Maastricht University. Registration of this trial was not required in the Netherlands as it is a nonmedical paper and is uncommon for psychological research such as this.

### Measurements

The survey used for the effect evaluation focused on determinants targeted by the e-coach and was based on the theoretical framework explaining teachers’ adoption and implementation of SRH developed by Paulussen et al [33], which is a combination of the theory of planned behavior [34], social cognitive theory [35], and diffusion of innovations theory [36]. Further description of and foundation for this framework can be found in a study conducted by Schutte et al [6]. At baseline (T0), we measured background characteristics of the teachers, including (SRH and LLL) teaching experience and their LLL curriculum-related beliefs and student response. At posttest (T1), we measured the same determinants but also included measures on completeness of and adherence to LLL implementation. In addition, subjective evaluations of the e-coach and the MHS training were included (this will be further discussed in the process evaluation) [31].

*Demographic variables* (T0) included gender, age, teaching subject, educational level of students, years of teaching experience, years of teaching SRH, perceived expertise in teaching SRH, perceived need for support in providing SRH, attitude toward teaching SRH, attitude toward reflecting on own SRH teaching methods, past experience with previous versions of LLL, and sexual morality.

Table 1 provides an overview of all *outcome measures*. For measuring *curriculum-related beliefs* (T0 and T1), *teacher benefits, subjective norms* *social support* *,* and *self-efficacy* were assessed together with *(anticipated) student responses.*

Teacher’s *implementation behavior* (T1) was measured based on rates of completeness of and adherence to LLL implementation. *Completeness* was expressed by the proportion of the 19 core learning activities of the LLL program being implemented (∑implemented activities/19×100). In this study, adherence was measured as one aspect or dimension of fidelity. 

**Table 1:** Measures, number of items, reliability, example items, and answer scale.

Measurements		Items	Cronbach alpha	Exemplary items (response scales)
**Demographic variables**				
	Gender	1		What is your gender? (0=female, 1=male)
	Age	1		What is your age?
	Teaching subject	1		What subject do you teach? (1=biology, 2=health care, 3=citizenship, 4=other)
	Years of teaching experience	1		How many years have you been working in education?
	Years teaching SRH^a^	1		How many years have you been teaching SRH?
	Perceived expertise teaching SRH	1		How experienced are you in teaching SRH? (1=very inexperienced, 7=very experienced)
	Perceived need for support in providing SRH	1		Do you need support in providing SRH lessons? (1=no, certainly not; 5=yes, certainly)
	Attitude toward teaching SRH	6	.82	Indicate what you think about teaching SRH: Teaching SRH is… (important, necessary, fun, difficult, comfortable, competent; 1=not at all, 7=yes totally)
	Attitude toward reflecting on own SRH teaching methods	1		Indicate what you think about reflecting on your own SRH teaching methods: (important, useful, good; 1=not at all, 7=yes, totally)
	Use of previous LLL^b^	1		Have you used the previous LLL in the past for SRH lessons? (0=no, 1=yes)
	Years teaching LLL	1		For how many years have you been using LLL?
	Sexual morality	5	.62	Young people who have just met should not have sex (1=strongly disagree, 5=strongly agree)
**Curriculum-related beliefs**				
	Teacher benefits	6	.72	I gained insight in the sexuality experience of youngsters (1=strongly disagree, 5=strongly agree)
	Subjective norms	6	.81	Do you think that the following people appreciate you using LLL to provide sexual education? (principal, governing body, external consultants or health education experts, students, colleagues teaching the same and colleagues teaching a different subject, parents; 1=no, certainly not; 5=yes, certainly)
	Social support	6	.75	Do you expect support from the following people when implementing LLL? (governing body, colleagues teaching the same and different subjects, and the parent association; 1=no, certainly not, 5=yes, certainly)
	Self-efficacy	12	.76	I am able to create a safe atmosphere in the classroom where students feel safe to openly talk about sex and relationships (1=no, certainly not; 5=yes, certainly)
**Interactive context**				
	Student response	3	.63	Indicate how students generally respond to LLL (interested, shy, positively; 1=not at all, 7=yes totally)
**E-coaching**				
	Used at all	1		Did you visit the “Lesgeven in de Liefde” website for teachers during your use of the new LLL program (0=no, never; 1=yes)
**Implementation behavior**				
	Completeness—calculated as percentage of the program (ie, learning activities) being implemented. (ie, Σlearning activities/ 19×100)	1		Did you cover this (learning activity)? (1=yes, 0=no)
	Adherence	1		How did you implement the new LLL program? (1=I reviewed the program and only selected a few ideas for my SRH lessons, 2=I reviewed the program and selected many ideas for my SRH lessons, 3=I used the program as a guideline for my lessons and delivered some lesson suggestions according to the teacher manual, 4=I followed the guidelines of the program as closely as possible and delivered most lesson suggestions according to the teacher manual, and 5=I delivered all lesson suggestions for the LLL program exactly according to the teacher manual)

^a^SRH: sexual and reproductive health.

^b^LLL: Long Live Love.

*Adherence* was measured as the extent to which the LLL program was implemented according to the guidelines as prescribed in the teacher manual, with scores ranging from 1 (“I reviewed the program and only delivered a few lesson suggestions according to the teacher manual) to 5 (“I delivered all lesson suggestions for the LLL program exactly according to the teacher manual”) [37]. All measures, including number of items, response scales, reliability, and exemplary items, are presented in Table 1.

### Analyses

Data were analyzed using IBM SPSS Statistics 24. Given the nested structure of the design and the data (partly repeated; measurements nested within teachers nested within schools), multilevel regression analyses were used to evaluate the effects of e-coaching on teachers’ implementation of LLL and its determinants. Unstandardized regression coefficients are reported, along with the standard error of beta. An additional advantage of the mixed regression is that it takes into account all participants, including those with only one measure. An unstructured covariance matrix for the repeated measures was selected. Two levels were defined in the multilevel analysis: school and teacher. The pseudo *R*^2^was calculated at school level and at teacher level for outcome variables measured only at posttest (completeness and adherence) and for outcome variables measured at pre- and posttest.

The model included the predictors group (1 for intervention group [e-coach] and 0 for control group) for the outcomes of implementation behavior (completeness and adherence), and group, time of measurement (baseline and posttest), and the interaction time × group for the determinants. The mixed model was estimated with the restricted maximum likelihood method to obtain unbiased variance estimates. The intraclass correlation coefficient was between .18 and .43, confirming that multilevel analysis was required.

## Results

### Participants Flow

**Figure 1:**
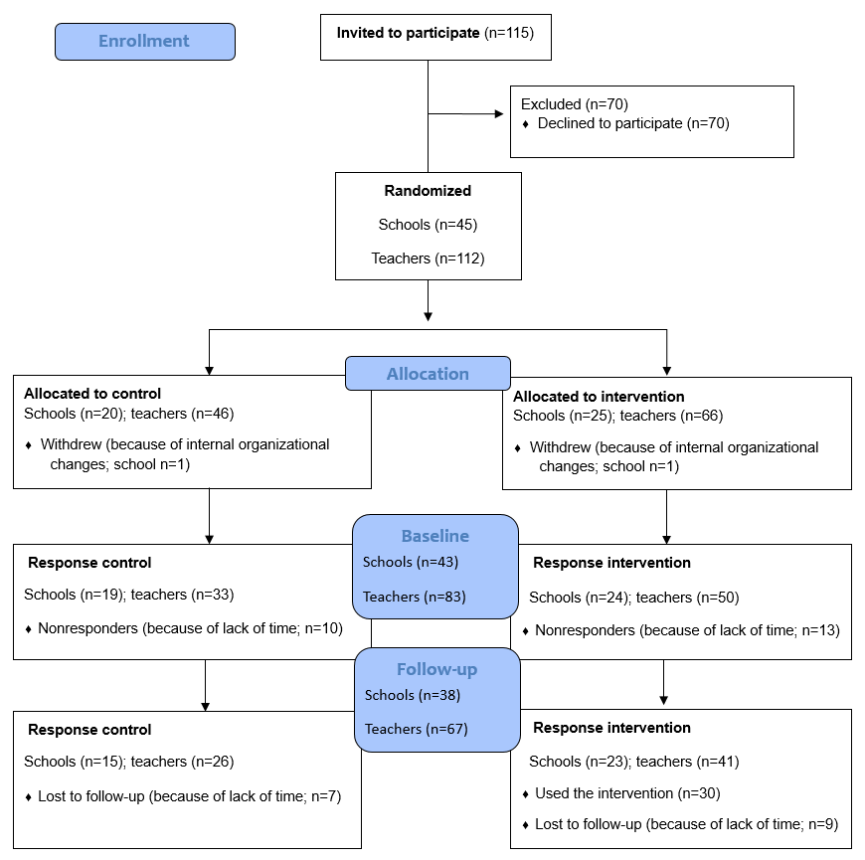
School allocation and participant flow.

Of the 115 schools approached, a total of 45 schools, including 112 teachers, agreed to participate in the pilot implementation of the revised LLL and the evaluation of their experience with implementing LLL. Teachers’ nonwillingness to participate was predominantly because of sexual education already having been provided in the school and lack of time. The schools were randomly assigned to either the waiting-list control group (20 schools including 46 teachers) or the e-coach intervention group (25 schools including 66 teachers). In addition, 2 schools (one from each condition, including 6 teachers) withdrew before the start of the pilot implementation of LLL because of internal organizational changes leaving 43 schools (106 teachers) at baseline. On average, there were 2 to 3 teachers per school. At baseline (T0), the survey was completed by 83 teachers (n=50 in the intervention group and n=33 in the control group) from 43 schools. Nonresponse was mainly because of lack of time. Follow-up measurement (T1) was completed by 67 teachers (80% of those completing T0; n=41 in the intervention group) from 38 schools. In addition, dropout at T1 (n=16) was mainly caused by lack of time. See Figure 1 for school allocation and participant flow. A dropout analysis, accounting for teachers’ background characteristics, indicated no significant differences between teachers who dropped out versus those who did not dropout (*t*_29-147_≤−0.36; *P*≥.07). Moreover, there was no significant difference in dropout between teachers in the intervention and control groups (χ^2^_1_=0.5; B (regression weight)=–.32; *P*=.48).

### Participants

Of the 83 teachers participating in the baseline questionnaire, 53 were female (64%) and 58 (70%) were biology teachers. The other teachers either taught the subject care (n=19; 22.89%) or citizenship (n=6; 7.23%). The mean age was 43 years. Years of teaching experience ranged from 1 to 39 years, whereas years of experience teaching sexual education ranged from 0 to 35. Teachers generally felt fairly experienced in teaching SRH, had a positive attitude toward teaching SRH, had a positive attitude toward reflecting on their own SRH teaching methods, had a positive attitude toward teaching SRH, and a permissive sexual morality. Teachers expressed a limited need for support in providing SRH lessons. One-third of the teachers had experience with the previous LLL program, ranging from 1 to 10 years. No differences could be observed at baseline between the intervention and control groups (see Table 2).

### Effects of E-Coaching on (Determinants of) Implementation of Long Live Love

Overall, teachers reported completing on average 73% of the LLL program (range 37%-98%), and 43% of teachers reported implementing the program largely in accordance with the guidelines in the teacher manual (mean 3.46, SD 0.75). Only 6% (n=4) implemented LLL exactly conforming to the guidelines in the teacher manual. No significant difference was found in completeness (regression weight=−2.12 [SE=3.99]; 95% CI −10.26 to 6.02; *P*=.60) or adherence (regression weight=0.09 [SE=.21]; 95% CI −0.33 to 0.51; *P*=.67) between teachers in the control group as compared with teachers in the e-coaching group, with small effect sizes (.17 and .14, respectively); see Table 3). In addition, no significant time × group interaction effect was found for the determinants of implementation behavior (*t*_67-75_≤0.69; *P*≥.22; see Table 4). All pseudo *R*^2^ values were smaller than .19 across all outcomes at both the school and teacher level. On the basis of the survey, it turned out that of the 41 teachers in the intervention group, 30 actually visited the website (75%). When comparing the 30 teachers who made actual use of the e-coaching website with the 37 teachers who did not, still no significant differences were found in completeness (regression weight=–2.21 [SE=3.62]; 95% CI for B −5.03 to 9.46; *P*=.54) or adherence of LLL (regression weight=0.06 [SE=0.19]; 95% CI for B −0.32 to 0.44; *P*=.74). No significant differences were found between determinants either (*t*_28-38_≤0.08; *P*≥.29).

**Table 2:** Demographic variables of teachers at T0.

Demographic variables (range)	Mean (SD): baseline (T0)		
	Total (n=83)	C-group^a^ (n=33)	I-group^b^ (n=50)
Age (23-64 years)	43.11 (11.38)	42.97 (11.30)	42.94 (11.59)
Years of teaching experience (1-39)	14.37 (10.43)	13.67 (10.86)	14.60 (10.08)
Years teaching SRH^c^ (0-35)	9.20 (7.91)	9.61 (7.73)	9.12 (8.09)
Experience teaching SRH (1-7)	4.63 (1.70)	4.85 (1.64)	4.50 (1.73)
Years teaching LLL^d^ (1-10)	4.80 (2.54)	4.44 (2.13)	5.21 (2.97)
Sexual morality (1-5)	2.33 (0.58)	2.28 (0.61)	2.38 (0.56)
Attitude toward teaching SRH (1-7)	5.87 (0.87)	5.86 (0.85)	5.87 (0.88)
Attitude toward reflecting on own SRH teaching methods (1-7)	5.94 (0.98)	6.03 (0.96)	5.86 (1.02)
Perceived need for support in providing SRH (1-5)	3.18 (1.04)	3.36 (.90)	3.08 (1.12)

^a^C-group: control group.

^b^I-group: intervention group.

^c^SRH: sexual and reproductive health.

^d^LLL: Long Live Love.

**Table 3:** Effect of e-coaching on teachers’ implementation behavior.

Implementation behavior	C-group^a^ (n=26), mean (SD)	I-group^b^ (n=41), mean (SD)	Regression weight B (SE^c^)	*P* value	95% CI for B
Completeness LLL^d^ (%)	73.85 (13.84)	72.35 (14.54)	–2.12 (3.99)	.60	−10.26 to 6.02
Adherence LLL (1-5)	3.38 (0.70)	3.51 (0.78)	0.09 (0.21)	.67	−0.33 to 0.51

^a^C-group: control group.

^b^I-group: intervention group.

^c^SE: standard error.

^d^LLL: Long Live Love.

**Table 4:** Effect of e-coaching on determinants of implementation behavior.

Determinants	Pretest		Posttest		Regression weight B (SE^a^)	*P* value	95% CI for B
	C-group^b^ (n=26), mean (SD)	I-group^c^ (n=41), mean (SD)	C-group (n=26), mean (SD)	I-group (n=41), mean (SD)			
Teacher benefits	3.61 (0.56)	3.57 (0.64)	3.21 (0.71)	3.08 (0.49)	−0.05 (0.16)	.77	−0.36 to 0.26
Subjective norms	4.08 (0.53)	4.03 (0.47)	4.24 (0.55)	4.09 (0.54)	−0.08 (0.15)	.60	−0.38 to 0.22
Social support	4.17 (0.44)	4.14 (0.57)	4.26 (0.55)	4.30 (0.55)	0.09 (0.15)	.54	−0.2 to 0.38
Self-efficacy	4.11 (0.33)	4.07 (0.39)	4.18 (0.47)	4.16 (0.42)	−0.02 (0.11)	.87	−0.23 to 0.19
Student response	5.71 (0.77)	5.51 (1.04)	5.37 (0.99)	5.17 (0.98)	0.03 (0.28)	.92	−0.53 to 0.58

^a^SE: standard error.

^b^C-group: control group.

^c^I-group: intervention group.

## Discussion

### Principal Findings

An e-coaching intervention was systematically developed to stimulate adherence and completeness of use of the revised Dutch secondary school-based sex education program LLL. The aim of this study was to improve teachers’ implementation behavior through self-reflection and skills development. The e-coaching was part of a broader implementation strategy that included a teacher training from the MHS before implementation.

Despite e-coaching being systematically developed, and with the input of experienced teachers, e-coaching was not found to be effective in changing teachers’ implementation behavior or its determinants. In general, teachers implemented the new LLL program moderately during the pilot study. The lack of effect could be a reflection of the intervention itself not being effective, either because of its development or implementation [18]. It is however often difficult to prove the effectiveness of interventions or implementation strategy even if they are solidly grounded in theory and evidence [18]. 

Several factors may explain the lack of effect of e-coaching on implementation of LLL. First, the study design itself may have had some flaws. Our study was a randomized controlled trial design and included a baseline measure and posttest; however, the limited number of teachers and schools at posttest could affect the generalizability of the results. In addition, there were 2 to 3 teachers per school, and this limited number may partly explain the poor implementation outcomes. Clustering practitioners using the same program within an organization, in groups of 3 or more, is usually considered an advantage in successful implementation [38]. Moreover, a “ceiling effect” could be present because of the implementation grade of teachers participating in the study already being high, making it difficult to improve using the e-coaching intervention. This suggests that e-coaching or another form of implementation enhancement may have been redundant in this particular case. Finally, the teachers who agreed to participate in the study may have been a biased sample of motivated, experienced teachers who were already capable of delivering LLL successfully. An additional methodological limitation in this study is the self-reported data [39]. Although this is often used to assess completeness and fidelity, methods of observation could perhaps further validate the results. Although the implementation strategy considered the individual teacher as well as the broader environment such as schools, MHS, and municipality, the focus was predominantly on individuals within these organizations. For example, no comparisons were made in implementation success of LLL and e-coach between schools. Considering the influence of decision-making processes in schools and organizations to influence top-down policy formation at the management level could strengthen sustainability of implementation [40].

Second, for an intervention to have an effect, it is important that the intervention is used and positively perceived. By not being used or insufficiently used by teachers, e-coaching is unlikely to have an effect [40-42]. Despite being designed to support teachers in their implementation of LLL, the website itself also needed to be effectively implemented. Teachers were perhaps not motivated to use the website because of their extensive experience in teaching SRH. Additionally, the broader implementation strategy developed to inform teachers about the e-coaching website was perhaps not optimally utilized, despite involvement of MHS professionals and teachers in the development process, potentially resulting in limited use and lack of effect of the website. For example, in this study, not all MHS provided a training and not all teachers who were offered a training accepted it. The broader implementation strategy could potentially be optimized to increase use of e-coaching by teachers. Motives for teachers’ use or nonuse of e-coaching need to be further explored, as well as means to increase use of the website by teachers. Taking contextual factors and individual factors into consideration remains important when stimulating implementation [6,18,29,43].

Finally, the intervention itself may have been suboptimal. The e-coach was aimed at determinants of completeness and fidelity but may not have addressed the exact needs of the target population, or been able to increase teachers’ awareness of the importance of completeness and adherence, or did not address completeness and adherence sufficiently or adequately. In developing e-coaching, program developers were already aware of the following challenges involved: (1) teachers did not see their suboptimal implementation behavior as problematic and (2) teachers expressed a minimal need for coaching during the interviews in the needs assessment phase of program development [28]. The developers attempted to address these challenges in the development of e-coaching by using an unobtrusive coaching technique and stimulating self-reflection, yet the question remained whether this would be successful or not. The e-coach intervention may have been unable to change teachers’ perceived need for coaching or change their awareness of their suboptimal implementation behavior with regard to completeness and adherence, which may be linked to a lack of effect. This re-emphasizes the importance of having a need for coaching or a desire for change before behavioral change [44]. Means of stimulating teachers to use the website need to be explored.

### Implications

Knowledge about implementation of Internet interventions and implementation of eHealth in the school settings particularly needs enhancement [45]. Website use was found to be related to factors associated with the visitor and the intervention website [46-48]. A large study in the Netherlands found that Information Technology use by teachers is limited. They either consult colleagues in their school for information or use the Internet mainly to find information, prepare their lessons, send emails to students, or give homework assignments and thus less for professional development [49]. Digital technologies are being increasingly used in the education system, bringing exciting opportunities for innovative ways of teaching and learning [50].

The strength of e-coaching is that, in addition to being aimed at specific determinants, it provides more than just a one-time training. Instead, it provides assistance during real-life implementation situations and has a longitudinal character in that teachers can visit the website as desired or need be [51]. Although completeness and adherence to program delivery are crucial to the effectiveness of the program, teaching quality of SRH lessons encompasses other teacher classroom-related skills, such as creating a safe and trusted environment, which form the conditions for providing these lessons. Such skills are addressed by e-coaching. In addition, other studies on providing sexual education have highlighted the importance of creating a safe environment when teaching this subject for optimal results [52,53]. “Teaching well” is thus more than completeness and fidelity. Therefore, in stimulating implementation of SRH programs, program developers should focus on enhancing completeness and adherence as well as supporting teachers in creating the classroom conditions that enable quality delivery of SRH lessons, as e-coaching has attempted to do.

### Conclusions

The lack of effect of e-coaching does not insinuate that Web-based coaching in itself is an ineffective strategy to promote adherence and completeness of program implementation, but in its current form, e-coaching is not the optimal instrument to achieve adherence and completeness of LLL specifically. To further understand why e-coaching had no effect and how it could potentially be improved, a process evaluation is required to find out how and to what extent teachers made use of the website, how they appreciated it, and what factors affected teachers’ use of the website.

## Appendix

Multimedia Appendix 1 CONSORT-EHEALTH checklist (V 1.6.1).

## Abbreviations

C-group: control group
I-group: intervention group
LLL: Long Live Love
MHS: municipal health services
SE: standard error
SRH: sexual reproductive health
